# Supplementary material for: O-Glycan-Altered Extracellular Vesicles: A Specific Serum Marker Elevated in Pancreatic Cancer
Source: Cancers (Basel). 2020 Aug 31;12(9):2469. doi: 10.3390/cancers12092469 (PMC7563872; doi:10.3390/cancers12092469)
Supplement: Supplementary file 1 [file cancers-12-02469-s001.pdf]

Supplementary Materials: *O*-Glycan-Altered Extracellular vesicles: A Specific Serum Marker Elevated in Pancreatic Cancer

Takahiro Yokose, Yasuaki Kabe, Atsushi Matsuda, Minoru Kitago, Sachiko Matsuda, Miwa Hirai, Tomomi Nakagawa, Yohei Masugi, Yuki Nakamura, Masahiro Shinoda, Hiroshi Yagi, Yuta Abe, Go Oshima, Shutaro Hori, Yutaka Nakano, Kazufumi Honda, Ayumi Kashiro, Chigusa Morizane, Satoshi Nara, Shojiro Kikuchi, Takahiko Shibahara, Makoto Itonaga, Masayuki Ono, Atsushi Kuno, Hiroshi Handa, Michiie Sakamoto, Makoto Suematsu, Yuko Kitagawa

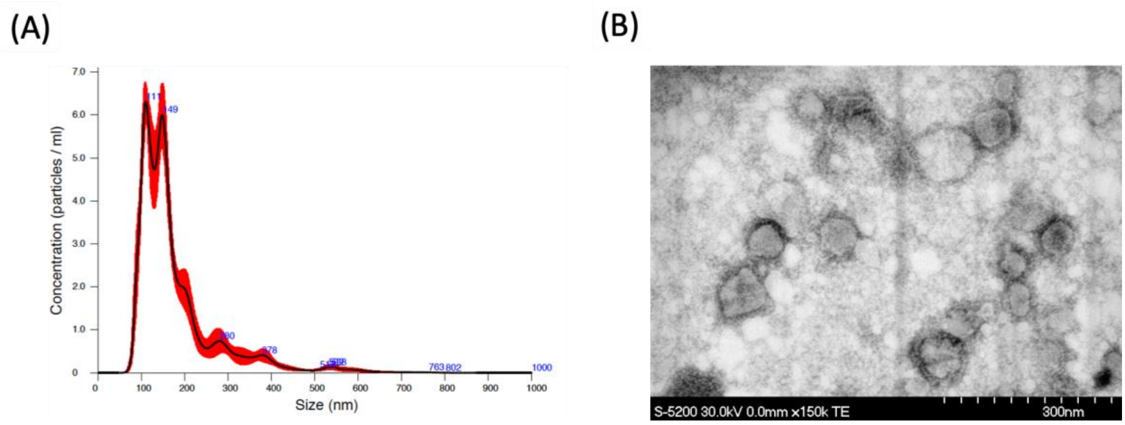

Figure S1. Validation of the EVs isolated from PC sera using Magcapture. (**A**) Size distribution of the isolated EVs analyses with NanoSight. (**B**) Validation of the isolated EVs analyses with STEM.

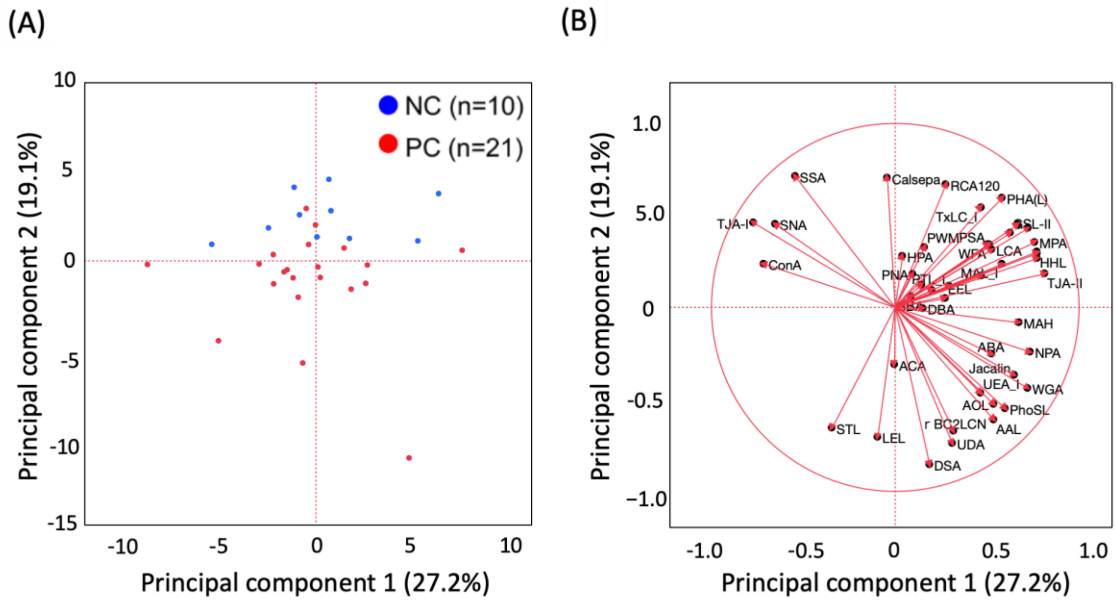

Figure S2. Profiling of the EVs containing specific glycans in PC sera with lectin microarray. EVs in PC (n = 21) or NC (n = 10) sera were isolated with Magcapture Exosome Isolation Kit and then labeled with Cy3-succimidyl ester. The labeled EVs were analyzed with lectin microarray immobilized with 45 lectins. The 2D plots shows the principal component analysis of glycan profiling. NC and PC are indicated in blue and red, respectively. Relative intensity of 45 lectins shown as a biplot.

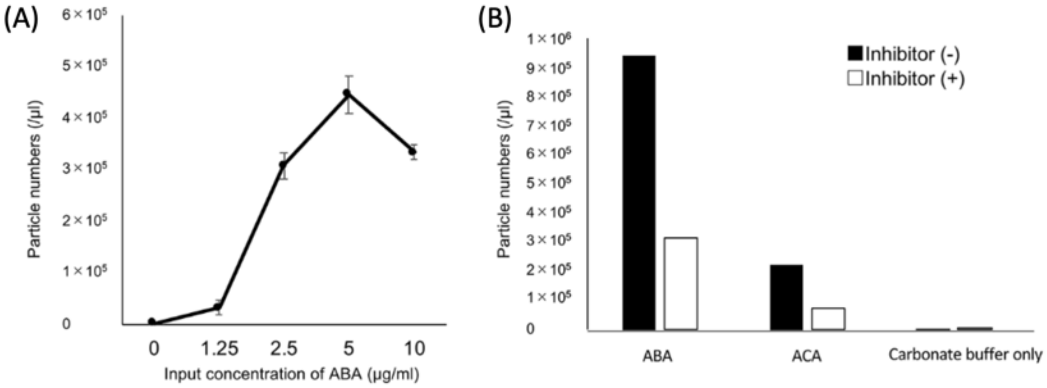

Figure S3. Optimization of ExoCounter system using lectin. (**A**) EVs in BxPC3 cell culture (n = 3) were analyzed using discs coated with ABA and anti-CD9 Ab-conjugated beads. The concentration of ABA lectin for disc coating was changed from 0 to 10 µg/mL. (**B**) EVs in BxPC3 cell culture medium were analyzed using discs coated with or without ABA or ACA and anti-CD9 Ab-conjugated beads with ExoCouter. Excess carbohydrates 0.2mM sialyl lactose or 0.2M lactose for inhibiting ABA or ACA, respectively, were added when blocking and injecting sample solutions into each well.

Table S1. Lectin microarray analysis.

Lectins	Study 1				Study 2		
	Mean normalized signals		Tumor / Normal ratio	p value	Mean normalized signals		Pre / Post operative ratio
	Pancreatic cancer	Normal control			Pre operative	Post operative	
LTL	0.002	0.001	1.894	0.324	0.006	0.004	1.692
PSA	0.233	0.242	0.965	0.655	0.321	0.365	0.878
LCA	0.390	0.434	0.898	0.186	0.519	0.636	0.816
UEA_I	0.018	0.016	1.088	0.857	0.037	0.022	1.667
AOL	0.103	0.105	0.981	0.952	0.196	0.119	1.654
AAL	0.506	0.452	1.119	0.443	0.595	0.538	1.107
MAL_I	0.079	0.101	0.779	0.220	0.119	0.092	1.294
SNA	4.636	4.827	0.960	0.489	3.765	3.144	1.197
SSA	4.961	6.052	0.820	<0.001	4.338	3.956	1.097
TJA-I	8.492	8.691	0.977	0.564	7.388	6.747	1.095
PHA(L)	0.048	0.094	0.508	0.030	0.094	0.078	1.201
ECA	0.061	0.072	0.842	0.573	0.123	0.148	0.828
RCA120	2.043	2.267	0.901	0.027	2.143	1.966	1.090
PHA(E)	0.898	1.103	0.814	0.085	1.165	1.214	0.959
DSA	2.746	1.997	1.375	<0.001	2.773	2.909	0.953
GSL-II	0.005	0.017	0.291	0.045	0.033	0.034	0.977

NPA	0.468	0.467	1.001	0.995	0.597	0.725	0.823
ConA	2.018	1.986	1.016	0.848	1.478	1.901	0.778
GNA	0.110	0.124	0.890	0.483	0.229	0.222	1.032
HHL	0.125	0.154	0.815	0.324	0.273	0.232	1.175
ACG	1.144	1.273	0.899	0.157	1.220	1.007	1.211
TxLC_I	1.378	1.805	0.764	0.009	1.980	1.944	1.019
BPL	0.007	0.012	0.615	0.282	0.024	0.016	1.521
TJA-II	0.522	0.578	0.904	0.354	0.828	0.720	1.150
EEL	0.010	0.008	1.149	0.863	0.023	0.021	1.090
ABA	0.707	0.570	1.239	0.063	0.683	0.492	1.387
LEL	2.837	2.223	1.276	0.001	2.976	3.436	0.866
STL	2.398	1.666	1.440	<0.001	2.193	2.666	0.822
UDA	2.891	2.471	1.170	0.011	3.266	4.190	0.780
PWM	0.004	0.006	0.675	0.622	0.016	0.020	0.796
Jacalin	0.796	0.739	1.077	0.533	0.802	0.683	1.174
PNA	0.000	0.001	0.488	0.643	0.000	0.000	0.867
WFA	0.003	0.017	0.154	0.191	0.015	0.004	3.576
ACA	0.522	0.435	1.199	0.058	0.535	0.455	1.175
MPA	0.062	0.099	0.624	0.040	0.139	0.094	1.470
HPA	0.000	0.001	0.000	0.343	0.000	0.000	ND
VVA	0.004	0.007	0.512	0.508	0.004	0.001	5.396
DBA	0.001	0.000	ND	0.148	0.000	0.000	ND
SBA	0.002	0.001	2.716	0.473	0.001	0.004	0.289
Calsepa	1.389	1.736	0.800	0.012	1.363	1.293	1.054
PTL_I	0.001	0.000	2.954	0.411	0.002	0.000	ND
MAH	0.133	0.117	1.142	0.438	0.167	0.143	1.170
WGA	0.909	0.804	1.131	0.322	1.027	1.062	0.968
rBC2LCN	0.008	0.001	9.813	0.237	0.016	0.013	1.217
PhoSL	1.333	1.230	1.084	0.439	1.527	1.682	0.908

ND, not determined

Table S2. Patient characteristics of cohort 1 and 2.

Parameter	Cohort 1		Cohort 2	
	PC (*n* = 68)	Control (*n* = 77)	PC (*n* = 49)	Control (*n* = 21)
Age, y [median (range)]	71(43–85)	49 (30–72)	68 (38–86)	53 (42–86)
Sex				
Male	37	19	26	9
Female	31	8	23	12
CA19-9, IU/mL [median (range)]	54 (1–2392)		186 (1–36800)	
Neoadjuvant treatment	0		0	
Tumor location				
Head	39			
Body or tail	29			
Primary tumor status				
T1	15			
T2	45			
T3	8			
Lymph node metastasis status				
N0	17			
N1	28			
N2	23			
Pathological stage				
IA	8		2	
IB	8		6	
IIA	0		0	
IIB	27		8	
III	21		10	
IV	4		23	

PC, pancreatic cancer; CA19-9, carbohydrate antigen 19-9

Table S3. The abbreviations and glycan binding specificity of 45 lectins immobilized onto LecChip used in this study

	Lectins	Origin	Binding specificity
1	LTL	*Lotus tetragonobus*	Fucα1-3GlcNAc, Sia-Le^x and Le^x
2	PSA	*Pisum sativum*	Fucα1-6GlcNAc and α-Man
3	LCA	*Lens culinaris*	Fucα1-6GlcNAc and α-Man , α-Glc
4	UEA-I	*Ulex europaeus*	Fucα1-2LacNAc
5	AOL	*Aspergillus oryzae*	Terminal α-Fuc, Sia-Le^x and Le^x
6	AAL	*Aleuria aurantia*	Terminal α-Fuc, Sia-Le^x and Le^x
7	MAL	*Maackia amurensis*	Siaα2-3Gal
8	SNA	*Sambucus nigra*	Siaα2-6Gal/GalNAc
9	SSA	*Sambucus sieboldiana*	Siaα2-6Gal/GalNAc
10	TJA-I	*Trichosanthes japonica*	Siaα2-6Galβ1-4GlcNAcβ-R
11	PHA-L	*Phaseolus vulgaris*	Tri-and tetra-antennary complex oligosaccharides
12	ECA	*Erythrina cristagalli*	Lac/LacNAc
13	RCA120	*Ricinus communis*	Lac/LacNAc
14	PHA-E	*Phaseolus vulgaris*	NA_2 and bisecting GlcNAc

15	DSA	*Datura stramonium*	(GlcNAc)$_n$, polyLacNAc and LacNAc (NA$_3$, NA$_4$)
16	GSL-II	*Griffonia simplicufolia*	Agalactosylated N-glycan
17	NPA	*Narcissus pseudonarcissus*	Non-substituted α1-6Man
18	ConA	*Canavalia ensiformis*	α1-6Man (inhibited by presence of bisecting GlcNAc)
19	GNA	*Galanthus nivalis*	Non-substituted α1-6Man
20	HHL	*Hippeastrum Hybrid*	Non-substituted α1-6Man
21	ACG	*Agrocybe cylindracea*	Siaα2-3Galβ1-4 GlcNAc
22	TxLC-I	*Tulipa gesneriana*	Man3 core, bi- and tri-antennary complex-type N-glycan, GalNAc
23	BPL	*Bauhinia purpurea*	Galβ1-3GalNAc and NA$_3$, NA4
24	TJA-II	*Trichosanthes japonica*	Fucα1-2Gal, β-GalNAc > NA3, NA4
25	EEL	*Euonymus europaeus*	Galα1-3[Fucα1-2 Gal] > Galα1-3 Gal
26	ABA	*Agaricus bisporus*	Galβ1-3GalNAcα-Thr/Ser (T) and sialyl-T
27	LEL	*Lycipersicon esculentum*	(GlcNAc)n and polyLacNAc
28	STL	*Solanum tuberosum*	(GlcNAc)n and polyLacNAc
29	UDA	*Urtica dioica*	(GlcNAc)n and polyLacNAc
30	PWM	*Phytolacca americana*	(GlcNAc)n and polyLacNAc
31	Jacalin	*Artocarpas integliforia*	Galβ1-3GalNAcα-Thr/Ser (T) and GalNAcα-Thr/Ser (Tn)
32	PNA	*Arachis hypogaea*	Galβ1-3GalNAcα-Thr/Ser (T)
33	WFA	*Wisteria floribunda*	Terminal GalNAc (e.g., GalNAc◎1-4GlcNAc)
34	ACA	*Amaranthus caudatus*	Galβ1-3GalNAcα-Thr/Ser (T)
35	MPA	*Maclura pomifera*	Galβ1-3GalNAcα-Thr/Ser (T) and GalNAcα-Thr/Ser (Tn)
36	HPA	*Helix pomatia*	Terminal GalNAc
37	VVA	*Vicia villosa*	α-, β-linked terminal GalNAc and GalNAcα-Thr/Ser (Tn)
38	DBA	*Dolichos biflorus*	GalNAcα-Thr/Ser (Tn) and GalNAcα1-3GalNAc
39	SBA	*Glycine max*	Terminal GalNAc (especially GalNAcα1-3Gal)
40	Calsepa	*Calystegia sepium*	Man, Maltose
41	PTL-I	*Psophocarpus tetragonolobus*	α-GalNAc and Gal
42	MAH	*Maackia amurensis*	Siaα2-3Galβ1-3[Sia◎2-6GalNAc] α-R
43	WGA	*Triticum aestivum*	(GlcNAc)n and multivalent Sia
44	BC2LCN	*Griffonia Simplicifolia*	Fucα1-2Gal (H type 1 or 3)
45	PhoSL	*Griffonia Simplicifolia*	Fucα1-6(Core fucose)

Binding specificities are summarized from LfDB (Lectin frontier Fatabase; http://riodb.ibase.aist.go.jp/rcmg/glycodb/LectinSerch)

© 2020 by the authors. Licensee MDPI, Basel, Switzerland. This article is an open access article distributed under the terms and conditions of the Creative Commons Attribution (CC BY) license (http://creativecommons.org/licenses/by/4.0/).
